# Phase Behavior and Polymorphism of Saturated and Unsaturated Phytosterol Esters

**DOI:** 10.3390/molecules25235727

**Published:** 2020-12-04

**Authors:** Eva Daels, Imogen Foubert, Zheng Guo, Wim Thielemans, Bart Goderis

**Affiliations:** 1 Research Unit Food and Lipids, Department of Microbial and Molecular Systems (M²S), KU Leuven, Campus Kulak Kortrijk, Etienne Sabbelaan 53 Box 7659, 8500 Kortrijk, Belgium; eva.daels@kuleuven.be; 2 Leuven Food Science and Nutrition Research Centre (LFoRCe), Kasteelpark Arenberg 20 Box 2463, 3001 Leuven, Belgium; 3 Agro-Biotechnology Science Lab, Department of Engineering, Aarhus University, Gustav Wieds Vej 10 building 3141, 01.18, 8000 Aarhus, Denmark; guo@eng.au.dk; 4 Sustainable Materials Lab, Department of Chemical Engineering, KU Leuven, Campus Kulak Kortrijk, Etienne Sabbelaan 53 Box 7659, 8500 Kortrijk, Belgium; wim.thielemans@kuleuven.be; 5 Polymer Chemistry and Materials, Department of Chemistry, KU Leuven, Celestijnenlaan 200f Box 2404, 3001 Leuven, Belgium; bart.goderis@kuleuven.be

**Keywords:** phytosterol esters, polymorphism, synchrotron X-ray diffraction, lipid crystallization, melting behavior

## Abstract

This study investigated how the physicochemical characteristics of phytosterol esters are influenced by the chain length and degree of unsaturation of the fatty acid ester moiety. Saturated and unsaturated phytosterol esters (PEs) were synthesized by the esterification of different types of fatty acids (stearic, palmitic, lauric, oleic, and linoleic acid) to β-sitosterol. The non-isothermal crystallization and melting behavior of the pure PEs were analyzed. It was proven by X-ray diffraction that saturated β-sitosteryl esters and β-sitosteryl oleate formed a bilayer crystal structure. The lamellar spacings of the bilayer structure decreased with decreasing fatty acid chain length and with an increasing degree in unsaturation. The degree of unsaturation of the fatty acid chain of the β-sitosteryl esters also influenced the type of subcell packing of the fatty acid moieties in the bilayer structure, whether or not a metastable or stable liquid crystalline phase was formed during cooling. Furthermore, it was found that the melting temperature and enthalpy of the β-sitosteryl esters increased with an increasing fatty acid chain length while they decreased with an increasing degree of unsaturation. The microscopic analyses demonstrated that β-sitosteryl oleate formed much smaller spherulites than their saturated β-sitosteryl analogues.

## 1. Introduction

Phytosterols are plant-based compounds with a chemical structure similar to cholesterol [1]. In the last two decades, phytosterols have gained much scientific and commercial interest due to their cholesterol-lowering activity [2,3,4,5]. Phytosterols can be esterified with fatty acids to obtain phytosterol esters (PEs) to enhance their solubility in lipid-containing food products such as margarines and spreads [6]. In this type of food products, the crystallization and melting behavior of lipids is of extreme importance because their macroscopic properties, such as spreadability, hardness, appearance and mouth feel strongly depend on it [7]. Fundamental research on the crystallization and melting behavior of PEs is however very scarce.

Some publications described the crystallization and melting behavior of different types of pure PEs as analyzed by differential scanning calorimetry (DSC), X-ray diffraction (XRD) or polarized light microscopy (PLM) [8,9,10,11,12,13,14]. Most of these reported the melting temperature and melting enthalpy of the PEs except for Kuksis and Beveridge (1960), who did not report the melting enthalpy since they only used PLM [8]. In addition to the melting behavior, the crystallization behavior of β-sitosteryl laurate was described by Vu et al. (2004) [10] and that of a commercial phytosterol ester mixture by Daels et al. (2017) [14]. However, difficulties arise when trying to compare and use the reported data of these different studies since different or poorly defined methods have been used to extract the melting temperatures and melting enthalpies from the DSC experiments. Furthermore, data inconsistencies are also due to differences in the purity of the PEs used in the different studies.

When comparing publications, it is clear that the analyzing technique has an influence on the measured melting temperature and enthalpy. Kuksis and Beveridge (1960) used PLM as the only analysis technique and they defined the melting point as the temperature at which the material became fully liquid and the field of polarized light turned dark [8]. All other authors used DSC to monitor the melting behavior of the PEs, however, except for Panpipat et al. (2013) [11] and Daels et al. (2017) [14], they did not specify how they calculated the melting temperature from the DSC melting curve, i.e., whether they used the peak temperature or the offset temperature of melting. Furthermore, differences in heating rate also have a critical influence. Kuksis and Beveridge (1960) reported that upon increasing the heating rate, the melting temperature of all the investigated PEs decreased [8]. Panpipat et al. (2013) used a heating rate of 2.5 °C/min for PEs containing saturated fatty acids and 1 °C/min for PEs containing unsaturated fatty acids, while the other authors used a heating rate of 5 °C/min [14] or 10 °C/min [11]. Moreover, next to the heating rate, the crystallization history prior to heating, which also influences the subsequent melting behavior, is different among the different publications.

Secondly, in addition to the analyzing technique and conditions, the purity of the PEs used in the different studies is also greatly divergent. The authors of the different studies synthesized the PEs on lab scale by either enzymatic [10,11] or by chemical [8,12,13] esterification reactions, except for Daels et al. (2017) who studied a commercial phytosterol ester mixture. The PEs in the different studies contained varying concentrations of reactants (free phytosterols and free fatty acids or fatty acid methyl esters). In general, the purity of the PEs is determined by the conversion of the esterification reaction and the yield of the ensuing purification process, if any. The PE purity reported in the publications was sometimes very high (>95%) [12,13,14], but it was rather low (50–99%) in the publication of Kuksis and Beveridge (1960) [8] and very low (8.5–40.3%) in the study of Vu et al. (2004) [10], or not even mentioned in other studies [9,11]. When the purity is not high enough, the melting temperature of the PEs could be increased by the presence of free phytosterols as their melting temperature is significantly higher than that of PEs [15]. The melting peak could also be decreased by the presence of free fatty acids or fatty acid methyl esters since their melting temperature is significantly lower than that of PEs [16]. Finally, only Daels et al. (2017) provided X-ray-based crystallographic information on PEs. From XRD measurements, they derived that the commercial phytosterol ester mixture simultaneously formed two different structures upon cooling: (i) a truly crystalline structure formed by palmitic and stearic esterified phytosterols and (ii) liquid crystals consisting mainly of phytosteryl oleate [14]. The commercial mixture consisted mainly of linoleic acid esterified phytosterols that did not crystallize in the observed time–temperature frame. As Daels et al. (2017) investigated a commercial phytosterol ester mixture, they could not present information on the phase behavior or crystal structures of the individual phytosterol esters.

The objective of this study was to investigate the influence of the chain length and the degree of unsaturation of the β-sitosterol ester fatty acid moiety on the non-isothermal crystallization and melting behavior of β-sitosteryl esters using DSC. The crystal structure of the β-sitosteryl esters was examined for the first time with time-resolved synchrotron XRD and insights into the microstructure were obtained using PLM. The β-sitosteryl esters containing different types of fatty acids (stearic (C18:0), palmitic (C16:0), lauric (C12:0), oleic (C18:1) and linoleic acid (C18:2)) were synthesized by enzymatic esterification. The β-sitosteryl esters contained no unreacted ingredients (free sterols or free fatty acids) as determined by thin layer chromatography (TLC).

## 2. Results and Discussion

### 2.1. Differential Scanning Calorimetry

**Figure 1:**
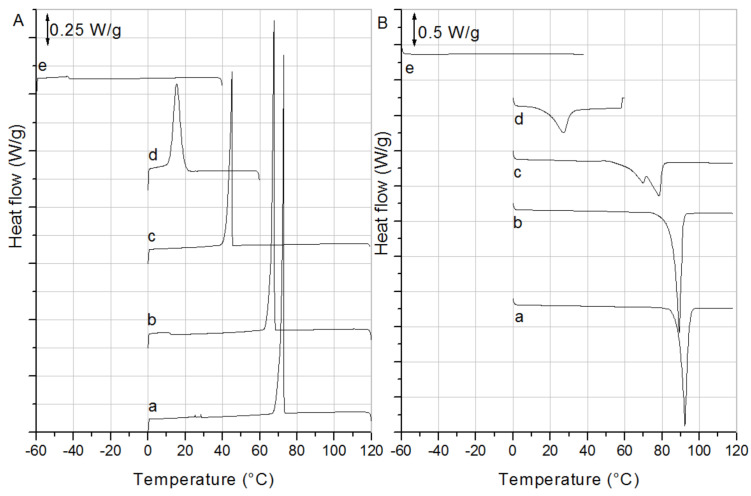
DSC cooling (**A**) and heating (**B**) run of the β-sitosteryl esters (a: P-stearate; b: P-palmitate; c: P-laurate; d: P-oleate; and e: P-linoleate).

The crystallization and melting curves of the β-sitosteryl esters are shown in Figure 1A,B, respectively, and the parameters obtained from them are listed in Table 1. The crystallization curves of the saturated β-sitosteryl esters were very similar and exhibited one sharp crystallization peak with the crystallization peak maximum (T_c_max_) only slightly different from the onset temperature of crystallization (T_c_onset_). The reproducibility of the crystallization curves of the saturated β-sitosteryl esters was rather low and decreased with the decreasing fatty acid chain length (results not shown). This is reflected in the increasing standard deviations on T_c_onset_ and T_c_max_ and is related to an inefficient primary nucleation as will be shown further by PLM. Nucleation is a stochastic process, which leads to poor reproducibility if only a few nuclei are involved that grow into rather large crystals. On the other hand, it seems that, once nucleated, crystal growth proceeds rather quickly as deduced from the sharpness of the exothermic transition. Occasionally, the sudden, massive exothermic heat associated with crystallization led to a rise in the temperature and a temporary looping of the DSC signal to higher temperatures (data not shown) [17].

The crystallization curves of the unsaturated β-sitosteryl esters differed from those of the saturated β-sitosteryl esters and were also mutually very dissimilar. The crystallization curve of β-sitosteryl oleate (P-oleate) contained one peak with a reproducible T_c_onset_ and T_c_max_. Therefore, it is expected that the nucleation efficiency of P-oleate was higher than that of the saturated β-sitosteryl esters resulting in more and smaller crystals (confirmed by XRD and PLM). The DSC crystallization curve of β-sitosteryl linoleate (P-linoleate) contained one very broad and weak peak which was barely visible resulting in a low reproducibility in T_c_onset_ and T_c_max_ determination.

The DSC melting curves of the saturated β-sitosteryl esters were more reproducible than their crystallization curves (results not shown) as reflected in the smaller standard deviations (Table 1). This is not surprising since in contrast to crystallization, melting is rarely nucleation controlled [18]. The heating runs of the β-sitosteryl esters contained one melting peak, except for the heating run of β-sitosteryl laurate (P-laurate) that contained two melting peaks, the cause of which was explained while discussing the small angle X-ray scattering (SAXS) and wide angle X-ray diffraction (WAXD) results (Section 2.3).

As was expected from the literature, the onset temperature of melting (T_m_onset_) and the melting enthalpy (ΔH_melt_) of the β-sitosteryl esters (Table 1) increased with an increasing fatty acid chain length because a longer fatty acid chain results in stronger intermolecular interactions and better packing [8,9,10,11,12,13]. On the other hand, T_m_onset_ and ΔH_melt_ decreased with an increasing degree in the unsaturation of the fatty acid chain and the effect was significantly bigger compared to that of the fatty acid chain length because the double bond in the fatty acid disturbs crystal packing to a greater extent [11].

The very low value of ΔT of the P-oleate and ΔH_melt_ of P-linoleate indicates that the crystals formed by these β-sitosteryl esters were probably not highly structured. As confirmed by XRD, indeed, rather than true crystals, liquid crystals were formed in these cases, which are known to exhibit little supercooling during formation. The ΔH_melt_ of P-oleate was much higher than that of P-linoleate indicating that next to the liquid crystalline phase, P-oleate also formed true crystals. This was also confirmed by XRD (Section 2.3).

### 2.2. Polarized Light Microscopy

**Figure 2:**
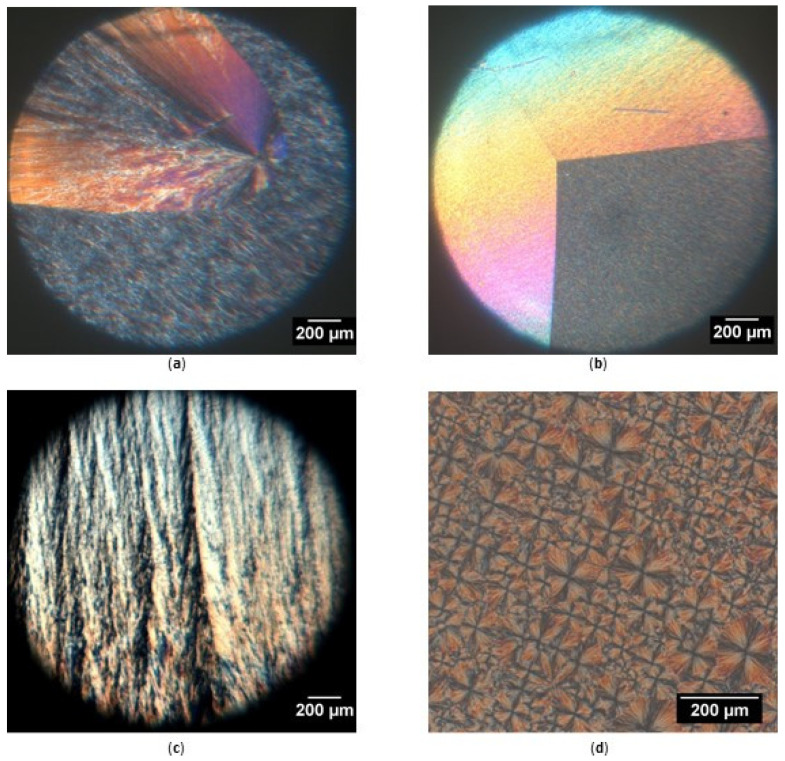
Polarized light microscopy image of spherulites of β-sitosteryl esters at the end of the cooling (0 °C). (**a**): P-palmitate; (**b**): P-laurate; (**c**): P-stearate; and (**d**): P-oleate. The white scale bar represents 200 µm. Images were taken at a magnification of 40×, except for image D which was taken at a magnification of 100×.

Figure 2 shows some images of the microstructure of the different β-sitosteryl esters. The microstructure formed by the saturated β-sitosteryl esters during cooling was very similar; each containing the different features that are shown in Figure 2a–c at different places in the sample. During cooling, only a few very large spherulites were formed as the one in β-sitosteryl palmitate (P-palmitate) shown in Figure 2a.

The growth rate of the saturated β-sitosteryl ester crystals was 50 ± 5 µm/s for β-sitosteryl stearate (P-stearate), 43 ± 6 µm/s for P-palmitate, and 56 ± 15 µm/s for P-laurate. The crystal growth rates of P-stearate, P-palmitate and P-laurate were about 600, 100, and 100 times faster than that of isothermally crystallizing tristearin [19], tripalmitin [20] and trilaurin [21], respectively, at a high degree of supercooling. However, as in the entire observation field, no more than one nucleus was observed, the nucleation rate of the saturated β-sitosteryl esters was too low to measure. Although the nucleation efficiency was low, the crystal growth rate of the saturated β-sitosteryl esters thus seemed to be very high. The combination of a low nucleation possibility and a high crystal growth rate resulted in the formation of very large crystals. The formed crystals were so large that it was only possible to see one spherulite in the observation field. As a consequence, using this setup it was not possible to determine the size of the spherulites of the saturated β-sitosteryl esters. It can only be stated that the spherulite diameter was larger than 2 mm, the diameter of the observation field.

The hyperbolic shape of some of the spherulites such as the one shown in Figure 2a could be explained by the fact that it was formed relatively late and was completely swallowed by an even larger spherulite created earlier [22]. At other places in the sample (e.g., Figure 2b), linear interfaces were also observed between the spherulites of the same size and growth rate. These were nucleated more or less simultaneously [22]. At places where no spherulites or interfaces were present, a fiber-like microstructure was observed reflecting the fiber-like arrangement of the nanocrystal aggregates in the spherulite (e.g., Figure 2c).

As shown in Figure 2d, the microstructure of P-oleate was clearly different to that of the saturated β-sitosteryl esters. In this case, the nucleation rate could be determined and was 1.8 ± 0.6 nuclei/mm^2^s. The crystal growth rate was 0.49 ± 0.16 µm/s, approximately 100 times lower than that for the saturated β-sitosteryl esters. Due to the low crystal growth rate relative to the nucleation rate, densely packed crystals of varying size were obtained at the end of cooling as shown in Figure 2d. With a spherulite diameter between 20 and 180 µm, the crystals were much smaller than those of saturated β-sitosteryl esters.

It is remarkable that the spherulites of saturated as well as unsaturated β-sitosteryl esters showed various colors such as yellow, orange, brown, light blue, green and pink. Since the microscope did not contain any type of filters, the crystal planes must have acted as a sort of filter causing destructive interference for certain wavelengths resulting in the interference colors observed through the crossed polarizers. The exact color observed at a certain position depends on many factors such as the degree of orientation of the crystal fragments in the radial direction of the spherulite nucleus, the degree of crystallinity, the distance of the position from the spherulite nucleus, and the thickness of the sample [23]. The grey and colored areas in Figure 2a,b are tentatively associated with different local crystal orientations with respect to the microscope polarizer axes. The colors were not further investigated as they are not really relevant here.

During the heating of the β-sitosteryl esters there was a small temperature range during which the colors faded and the image became greyish and eventually turned completely black. No other changes in the microstructures were observed.

### 2.3. X-ray Diffraction

**Figure 3:**
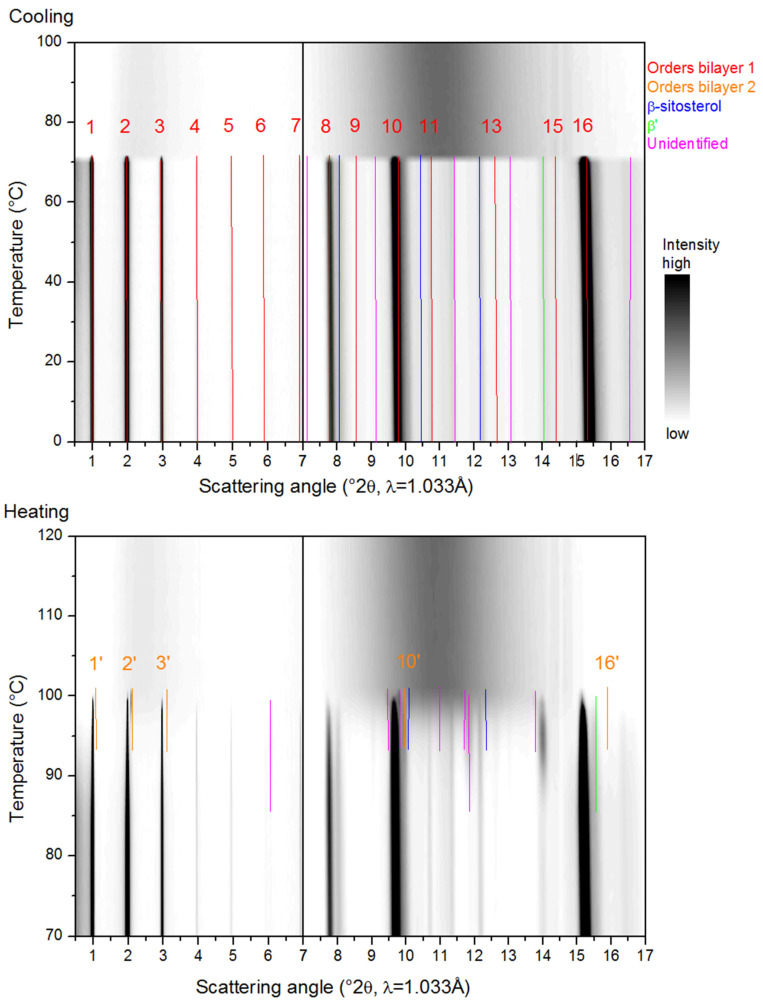
Small angle X-ray scattering (SAXS) (left) and wide angle X-ray diffraction (WAXD) (right) data of P-stearate during cooling (**upper image**) and heating (**lower image**). The legend indicates the identification of the spacings as an order reflection of bilayer 1 or bilayer 2, or as related to the packing of β-sitosterol moieties (β-sitosterol) or the β′ subcell packing of the stearic acid moieties (β′) or unidentified (UI).

**Figure 4:**
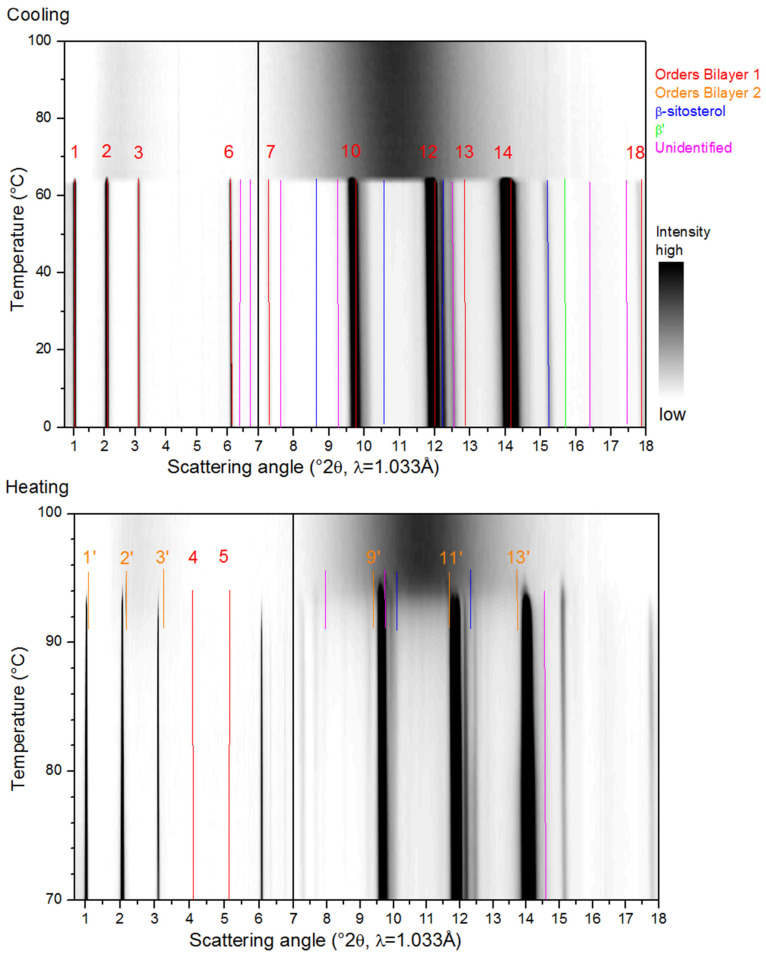
Small angle X-ray scattering (SAXS) (left) and wide angle X-ray diffraction (WAXD) (right) data of P-palmitate during cooling (**upper image**) and heating (**lower image**). The legend indicates the identification of the spacings as an order reflection of bilayer 1 or bilayer 2, or as related to the packing of the β-sitosterol moieties (β-sitosterol) or the β′ subcell packing of the palmitic acid moieties (β′) or unidentified.

**Figure 5:**
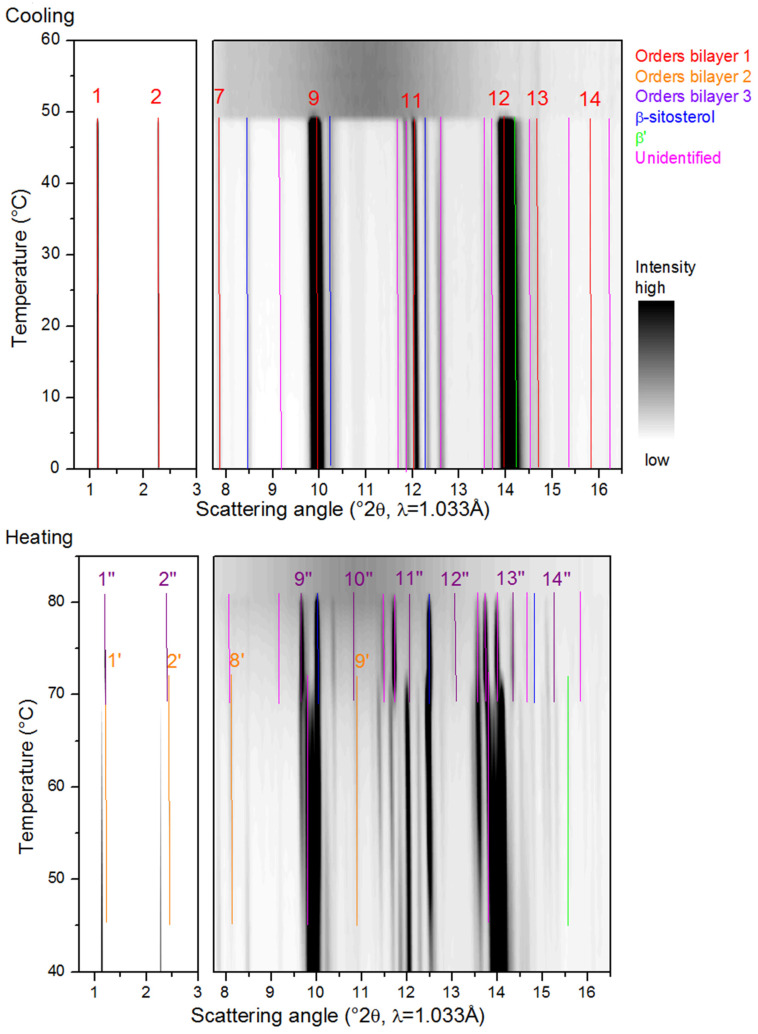
Small angle X-ray scattering (SAXS) (left) and wide angle X-ray diffraction (WAXD) (right) data of P-laurate during cooling (**upper image**) and heating (**lower image**). The legend indicates the identification of the spacings as an order reflection of bilayer 1, bilayer 2 or bilayer 3, or as related to the packing of the β-sitosterol moieties (β-sitosterol) or the β′ subcell packing of the lauric acid moieties (β′) or unidentified.

**Figure 6:**
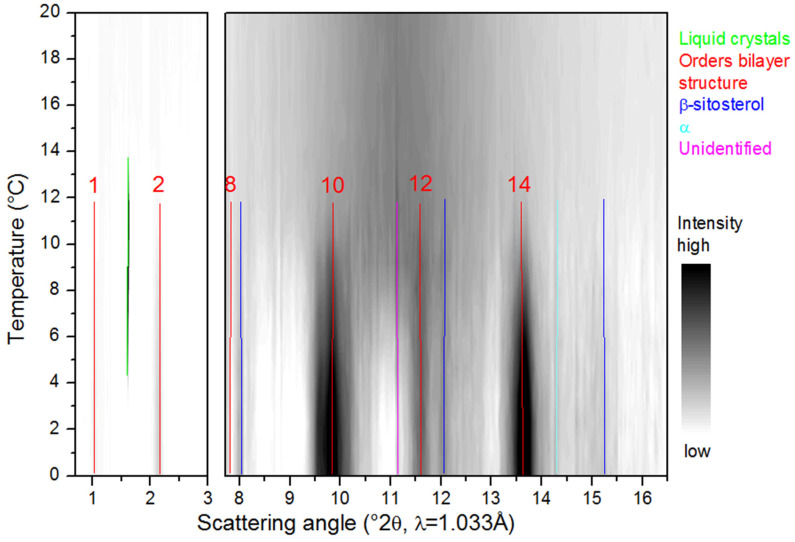
Small angle X-ray scattering (SAXS) (**left**) and wide angle X-ray diffraction (WAXD) (**right**) data of P-oleate during cooling. The legend indicates the identification of the spacings as an order reflection of the bilayer structure or as related to the packing of the β-sitosterol moieties (β-sitosterol), the α subcell packing of the oleic acid moieties (α) or the liquid crystals or unidentified.

**Figure 7:**
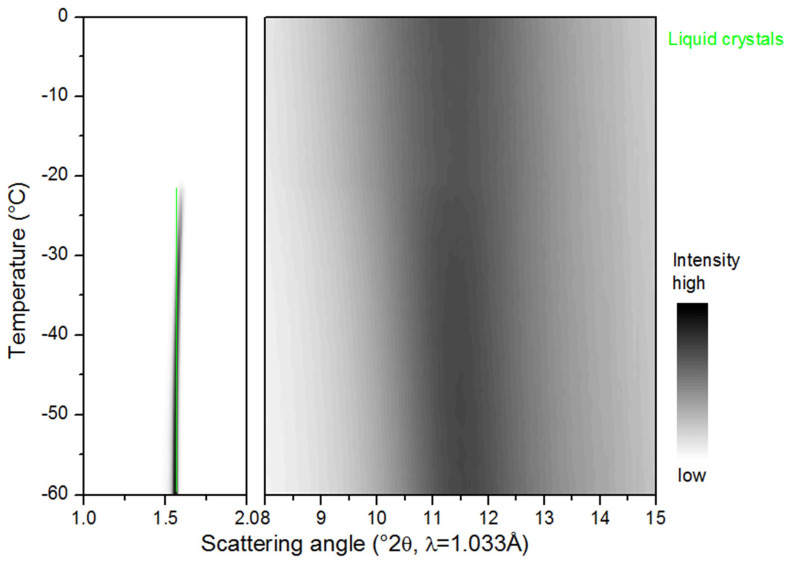
Small angle X-ray scattering (SAXS) (**left**) and wide angle X-ray diffraction (WAXD) (**right**) data of P-linoleate during cooling. The spacing related to the liquid crystals is marked in green.

Figure 3, Figure 4, Figure 5, Figure 6 and Figure 7 show the SAXS and WAXD patterns of the different β-sitosteryl esters during cooling and heating. The SAXS and WAXD peaks that were newly formed during cooling and heating are marked and their origin and spacings are listed in Table 2, Table 3, Table 4 and Table 5. The tables also indicate the temperature range in which the listed spacings emerged. The temperatures at which crystallization started, as indicated in Table 2, Table 3, Table 4 and Table 5, deviated somewhat from the values of T_c_onset_ listed in Table 1. The reason for this is the low reproducibility of the nucleation of the crystallization for the saturated β-sitosteryl esters, a slightly higher cooling rate (6 °C/min in the XRD analysis compared to 5 °C/min in the DSC analysis) due to a technical error of the Linkam device for P-oleate, and the broad shape of the crystallization peak in the DSC cooling run for P-linoleate.

#### 2.3.1. Cooling

**Figure 8:**
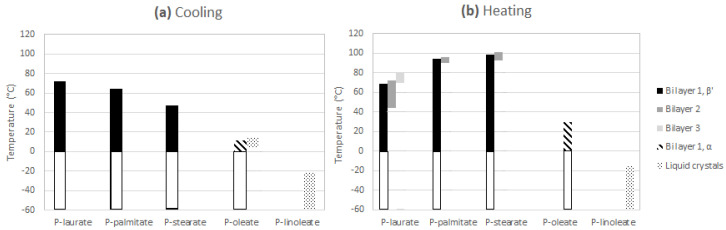
Morphology map highlighting the temperature ranges based on the SAXS and WAXD data in which the different phases exist during the cooling (**a**) and heating (**b**) of the different β-sitosteryl esters. White outlined temperature ranges were not examined. It is expected, however, that the structures at 0 °C remain within the uninvestigated low temperature ranges.

During the cooling of the saturated β-sitosteryl esters, strong reflections were observed in the SAXS patterns. These strong reflections were (almost all) linked to the same periodicity indicating the formation of a layered crystal structure as shown in Figure 3, Figure 4 and Figure 5 and Table 2, Table 3 and Table 4. The data in these figures and tables are summarized in a morphology map showing the temperature ranges in which the different β-sitosteryl esters structures are formed during cooling and heating (Figure 8). As many of the observed WAXD peaks were orders of the same periodicity, this indicates that the ordering of the molecules into the layered crystal structure was so strong that the lamellar reflections were also visible throughout the WAXD. Moreover, these periodicity peaks were the strongest peaks in the XRD patterns, confirming that the β-sitosteryl esters were highly ordered into the layered crystal structure. Similarly, as for the saturated β-sitosteryl esters, the SAXS and WAXD patterns of P-oleate also contained multiple orders of the same periodicity. From the XRD spacings related to the layered crystal structure, it could be derived that the saturated β-sitosteryl esters and P-oleate crystallized in a bilayer structure during cooling, as further discussed in Section 2.4.

We also examined whether the fatty acid moieties of the β-sitosteryl esters formed the same types of subcell packing as those observed in triglycerides. This was done by comparing the WAXD spacings in the XRD patterns of the β-sitosteryl esters with the typical short spacings of the aliphatic subcell packing, thereby taking into account their relative intensities as far as this could be justified (see below when discussing orientation effects). The identification of the subcell packings in the WAXD patterns was, however, disturbed by the presence of order peaks of the bilayer structure, often with a high intensity, at typical subcell spacings. For example, the spacing at 4.20 Å in the WAXD pattern of P-stearate could indicate a β′ subcell packing, but the second typical β′ peak is expected at the same spacing as the strong 16th order reflection of the bilayer structure at 3.84 Å [24]. However, the formation of a β′ subcell during cooling was confirmed by the WAXD patterns during heating in which the β′ peak at 3.82 Å was split off from the 16th order reflection. The situation for P-laurate was similar where the β′ spacing at 4.20 Å already emerged during cooling, but the other β′ spacing at 3.79 Å only became visible during heating when it was split off from the 16th order reflection. In P-palmitate, the formation of the β′ subcell was not confirmed as only the β′ spacing at 3.77 Å was visible, while the second β′ spacing coincided with the strong 14th order reflection which was not split into two peaks during cooling. The relative intensities of the β′ peaks could thus not be examined. While in P-stearate and P-laurate (and possibly also in P-palmitate), a β′ subcell packing was observed, and the oleic acid moieties of P-oleate were packed in the α polymorph as indicated by the spacing at 4.14 Å [24]. The α polymorph is required for P-oleate to overcome the steric hindrance between the oleic acid chains due to the presence of the double bond. It is known that the hexagonal packing of the α subcell structure has a lower packing density than the orthorhombic packing of the β′ subcell structure. No other types of subcell packings were identified in the XRD patterns of the β-sitosteryl esters because not all required spacings were present, and judging relative peak intensities was considered not reliable because of orientation effects (see further below).

Of the other remaining SAXS and WAXD spacings, some could be related to the ordering of the β-sitosterol moieties as they were very similar (except for a difference of 0.1 Å) to the spacings of pure β-sitosterol measured at room temperature that are reported in the literature [25,26].

Another type of structure that was formed only by P-oleate and P-linoleate were liquid crystals, as indicated by the presence of one very strong SAXS peak with no corresponding WAXD peaks (see Figure 6 and Table 5). During cooling, P-oleate first formed a liquid crystalline phase which then transformed into a more stable crystalline phase. The formation of the liquid crystalline phase and its transformation to a truly crystalline phase could, however, not be distinguished in the DSC melting curve in Figure 1. A possible explanation for this is that the formation of the liquid crystals and the formation of the true crystals were both included in the single DSC melting peak or that the heat released by the formation of the liquid crystals was smaller than the detection limit of the DSC. During the cooling of P-linoleate, the SAXS spectrum shows one peak with a very high intensity at the same spacing as for P-oleate, while the WAXD pattern does not show any peak formation (see Figure 7 and Table 5). Leeson et al. (2002) also reported the formation of a mesophase in P-oleate, which transformed to a more stable crystalline phase at a lower temperature and the formation of a stable mesophase in P-linoleate, but this was not based on XRD analyses [9]. A metastable mesophase with a very similar SAXS spacing is also formed by cholesteryl oleate [27].

Some of the spacings in the XRD patterns of the β-sitosteryl esters seemed not to be related to the bilayer structure, the packing of the β-sitosterol moieties, the subcell packing of the fatty acid moieties, nor the formation of liquid crystals. These spacings, which are indicated as ‘unidentified’ in Figure 3, Figure 4, Figure 5 and Figure 6 and Table 2, Table 3, Table 4 and Table 5, could have various possible origins. First, they may be related to the packing of phytosterol moieties other than β-sitosterol, as the β-sitosterol used for the synthesis of the β-sitosteryl esters also contained campesterol and β-sitostanol. The lack of the double bond in the sterol ring of β-sitostanol and especially the presence of a methyl group instead of an ethyl group in the sterol side chain of campesterol could lead to a somewhat different packing of the phytosterol moieties in the crystal structure resulting in different reflections [1]. Further research and XRD analysis of β-sitostanol and campesterol is needed to confirm this since this information is not available in the literature. Furthermore, these ‘unidentified’ spacings may also be related to the intermolecular fatty acid–phytosterol interactions, but a profound crystallographic study, which was out of the scope of this research, is necessary to investigate this hypothesis.

**Figure 9:**
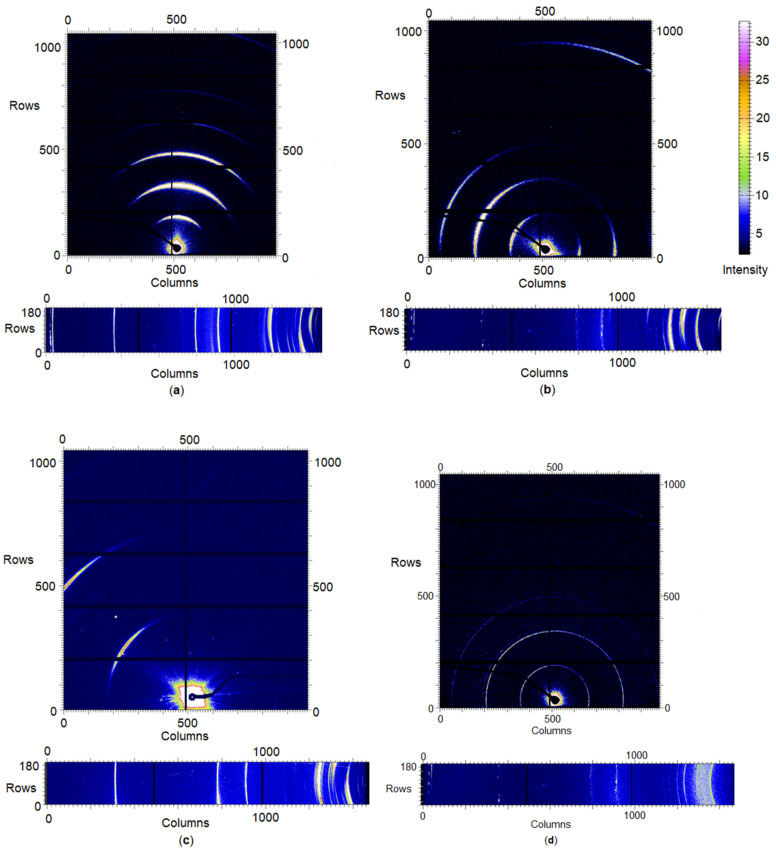
Two-dimensional (2D) patterns of the raw SAXS (above) and WAXD (below) data recorded at the end of the cooling stage (0 °C) of P-stearate (**a**), P-palmitate (**b**) and P-laurate (**c**) and during the heating of P-stearate at 93.6 °C (**d**). The direct beam, covered by the beam stop, is visible centrally in the SAXS arcs and rings. For the WAXD patterns, the direct beam is situated outside of the images on the right-hand side.

Finally, it is of interest to note that rather than complete Debye–Scherrer rings, incomplete diffraction arcs were observed in the 2D SAXS and WAXD patterns of the saturated β-sitosteryl esters prior to azimuthal averaging, as shown in Figure 9a–c. Such patterns resemble those of the fiber-like arrangement of the crystallites [28] and in general are due to preferred orientations. In the present case, PLM experiments demonstrated that the material crystallizes into very large spherulitic nanocrystal aggregates that are much bigger than the X-ray beam. The X-ray beam probes only a fragment of the spherulite, far from its nucleus where a nanocrystalline bundle consisting of parallel oriented nanoplatelets emanates radially from the spherulite nucleus, leading to diffraction arcs. This effect of preferred orientation may lead to the absence or weakening of some crystalline reflections and hence a different azimuthally averaged pattern compared to when perfect powders would have been studied. Arcs in the SAXS and WAXD region with a common azimuthal orientation are crystallographically related. Such anisotropy in XRD patterns and its origin have been described by Marangoni et al. (2012) [28] and Ueno et al. (2008) [29]. The arc patterns were different for the different β-sitosteryl esters. In the 2D patterns of P-palmitate, pairs of arcs were observed at opposite sides of the 2D pattern indicating that the X-ray beam probed the interface between the two spherulites. The 2D patterns of P-laurate showed smaller arcs than those of P-stearate, indicating that the P-laurate spherulite fragment was probed at a distance further away from the primary nucleus [29].

#### 2.3.2. Heating

The various changes in the XRD patterns of the saturated β-sitosteryl esters during heating were divided into two major events. First, the intensity of some of the SAXS and WAXD peaks increased from a temperature similar to T_m_onset_ and from this temperature, more SAXS and WAXD spacings became visible. Some of these apparently new spacings were related to the packing of the β-sitosterol moieties or the packing in the bilayer structure while others could not be identified. It was also noticed that approximately, from T_m_onset_, the 2D patterns left their arc-like shape and converted to full circles towards the end of the melting, as shown in Figure 9d. It is suggested that the least stable nanocrystallites (because of size or internal packing) melt and release crystal fragments from the spherulites that assume a fully random orientation. This random arrangement of the crystal fragments led to fully symmetric 2D patterns [28].

A second event in the SAXS and WAXD patterns of the saturated β-sitosteryl esters was the emergence of new peaks at a later stage during heating due to the transition to a new crystal structure. As indicated by the presence of the dimensions of the long spacings (and its higher orders in SAXS and WAXD) corresponding to the same periodicity, the new crystal structure was also a bilayer. Therefore, the bilayer structure formed during cooling is referred to as bilayer 1 while the bilayer structure formed during heating is referred to as bilayer 2. The long spacings of bilayer 2 were shorter than those of bilayer 1, probably indicating that the layer-to-layer packing had increased while the molecule-to-molecule packing decreased because of thermal expansion, thereby allowing a denser interdigitated layer-to-layer packing. In P-laurate, during heating, even a second structural transition occurred to yet another new crystal structure, which according to the d-spacings of the stack periodicity, also corresponded to a bilayer structure (bilayer 3). The layer-to-layer packing density in bilayer 3 was intermediate between that of bilayer 1 and bilayer 2.

Next to new order reflections related to the lamellar packing in bilayer 2 (and 3), also other new WAXD spacings emerged from the same temperature. Some of these other new WAXD spacings were very similar (except for a difference of 0.1 Å) to the spacings of pure β-sitosterol measured at room temperature that are reported in the literature [25,26], while none of them were related to fatty acid subcell packings. The bilayer structures formed by the saturated β-sitosteryl esters during heating were therefore probably dominated by the packing of the β-sitosterol moieties rather than the fatty acid moieties. Furthermore, just as for bilayer 1, some of the spacings of bilayer 2 (and 3) could not be identified.

During the heating of the unsaturated β-sitosteryl esters, no structural changes occurred as no new peaks occurred in SAXS or WAXD. The spacings related to the structures formed during the cooling of P-oleate and P-linoleate fully disappeared at 29.8 and −15.9 °C, respectively.

### 2.4. Characterization of the Crystal Structures

#### 2.4.1. The Bilayer Structure

**Figure 10:**
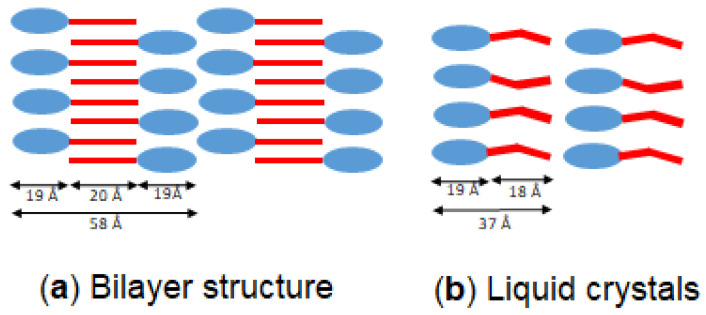
Bilayer structure of the β-sitosteryl esters formed by the saturated β-sitosteryl esters and P-oleate (**a**) and the structure of the liquid crystals formed by the unsaturated β-sitosteryl esters (**b**).

To date, and to the best of our knowledge, the XRD data of PEs have not been described in the literature, let alone the crystal structures of PEs. Except for an extra ethyl group in the sterol side chain, the molecular structure of the β-sitosteryl esters in this study are the same as the analogous cholesterol esters. Therefore, it could be expected that they form similar crystal structures. Cholesteryl laurate, palmitate, and stearate crystallize in a bilayer structure [27,30]. Only cholesteryl oleate is reported to form a monolayer structure when crystallizing [31]. It could be derived from the XRD data that P-stearate, P-palmitate and P-laurate are packed in a bilayer structure just like their analogous cholesterol esters. The presumed packing of the β-sitosteryl esters in the bilayer structure is based on the bilayer structure formed by cholesteryl esters and is presented in Figure 10a. The β-sitosterol moieties of the β-sitosteryl ester molecules in the bilayer packing are represented with a blue circle while the fatty acid moieties are indicated with a red bar. Two β-sitosteryl ester molecules form the asymmetric unit of the bilayer and are arranged in an antiparallel configuration.

It is possible to estimate the size of a β-sitosterol ester molecule, for example, P-stearate. Taking into account an average bond length of 1.50 Å and an average angle of 115° between the carbon atoms in the fatty acid chain, the length of a stearic acid moiety in P-stearate would be about 20 Å [32]. Assuming that the length of the β-sitosterol moiety is the same as that of a cholesterol molecule (being 19 Å) [33], which makes the size of one P-stearate molecule about 39 Å. The bilayer thickness of the P-stearate crystals is 59.2 Å as shown by the SAXS data, which is smaller than two times the size of a P-stearate molecule. This indicates that the stearic acid chains in the asymmetric unit of the bilayer interdigitate and interact with each other. This type of configuration in the bilayer also allows some space between the packed β-sitosterol moieties avoiding steric hindrance between them. The β-sitosterol rings of adjacent molecules are thus packed roughly in parallel to each other but the ring–ring interactions are not the dominant stabilizing force in this crystal form. On the other hand, the fatty acid chains are packed in an antiparallel configuration in a specific type of subcell packing. With this configuration, the theoretical lamellar size of the P-stearate bilayer equals 58 Å (being two times the size of a β-sitosterol moiety plus that of the stearic acid moiety as represented in Figure 10a), which is very similar to the experimentally observed length of the bilayer packing in the SAXS spectrum.

The same calculation can be made for the bilayer structure formed by the other β-sitosteryl esters. In Section 2.3, it was demonstrated that the SAXS spacings of the other saturated β-sitosteryl esters were 56.9 Å for P-palmitate and 51.9 Å for P-laurate (compared to 59.2 Å for P-stearate). With a decreasing fatty acid chain length, the SAXS spacings of the β-sitosteryl esters and thus also the lamellar spacing in the bilayer decreased. The SAXS spacing of P-oleate was 57.5 Å, which is also lower than that of P-stearate. An increase in the degree of the unsaturation of the fatty acid results in a decrease in the length of the fatty acid, because the double bond length is 1.37 Å and the double bond angles are 120° and 124° [31]. Therefore, the lamellar spacings in the bilayer structure and thus also in the SAXS spacings of P-oleate are shorter than those of P-stearate. Despite the kink in their chain, the oleic acid moieties could also interdigitate and were ordered in a subcell packing most probably because the oleate chains can be almost straight. Similarly, Craven et al. (1979) also described a straight conformation of the oleate chains of cholesteryl oleate molecules packed in a monolayer structure, which was explained by the torsion angles. According to them the oleate chains of cholesteryl oleate consisted of two sections of an almost planar zig-zag chain and a kink section with the double bond in between. The long axes of the two chain sections were almost parallel but with a relative displacement of 0.5 Å [31].

#### 2.4.2. The Liquid Crystal Structure

The SAXS spacings of the liquid crystal structures formed by P-oleate and P-linoleate were very similar to the size of the PE molecules indicating that the liquid crystal structure consists of a monolayer structure as shown in Figure 10b. In the liquid crystal structure, the fatty acid chains do not interdigitate and do not have a crystalline subcell packing.

## 3. Materials and Methods

### 3.1. Materials

β-sitosterol (≥70%) and oleic acid (≥90%) were purchased from Sigma-Aldrich (Brondby, Denmark). Linoleic acid (97%) was obtained from TCI Europe (Zwijndrecht, Belgium) and stearic acid (97%), palmitic acid (98%) and lauric acid (99%) from Acros Organics (Geel, Belgium). β-sitosterol and oleic acid were analyzed for their chemical composition since their purity was lower than that of the other starting materials. Table 6 shows the sterol composition of β-sitosterol as measured by the method described in Ryckebosch et al. (2012a) [34]. Table 7 reports the fatty acid composition of oleic acid as determined by the method described in Ryckebosch et al. (2012b) [35].

### 3.2. Enzymatic Synthesis of the β-Sitosteryl Esters

The synthesis of a series of fatty acid esters of β-sitosterol was based on the method of Panpipat et al. (2013) [36]. β-sitosterol was blended with a fatty acid (C12:0, C16:0, C18:0, C18:1 or C18:2) at a mole ratio of 1.0:1.0 (mol/mol) at a concentration of 0.2 M in 800 mL hexane. Immobilized *Candida antarctica* lipase A (Codexis, Redwood City, CA, USA) was added to the blend in the amount of 20% (wt% of β-sitosterol).

The reaction was conducted in a 1 L closed reactor under continuous mechanic stirring at 50 °C for 48 h in the presence of 62.5% (wt% of β-sitosterol) molecular sieves (Sigma-Aldrich, Brondby, Denmark) to remove water from the solvent. The reaction was terminated by filtering out the immobilized lipase and the molecular sieves by vacuum filtration. The residue was washed with hexane to remove any substrate or product. Five types of β-sitosteryl esters were obtained: P-laurate, P-palmitate, P-stearate, P-oleate, and P-linoleate.

A first, a rough estimate of the yield of the esterification reaction was obtained by TLC. Ten microliters of the resulting reaction mixtures was applied to a TLC plate together with 10 µL of β-sitosterol and 10 µL of each of the fatty acids as a reference. The plates were developed with two different developing solvents: first with chloroform/methanol/water (64:10:1, *v*:*v*:*v*) up to half of the plate and subsequently with chloroform/methanol/acetic acid (97.5:2.5:1, *v*:*v*:*v*) to nearly the top of the plate. In between the two solvents, and at the end, the plates were dried. The visualization of the spots on the plate was done by immersing the plate shortly in a sulfuric acid solution (30% in methanol) and subsequently heating the plate at 100 °C for 1 min with a blow dryer. The spots of β-sitosterol and of the β-sitosteryl esters showed a purple color, while those of the fatty acids were white. The retention factors (Rf) of β-sitosterol, the fatty acids, and the β-sitosteryl esters were 0.63, 0.47 and 0.91, respectively. The reaction yield was estimated based on the relative size of the β-sitosterol spot to the β-sitosteryl ester spot. For each type of β-sitosteryl ester, the reaction yield was clearly lower than 90% and was estimated to be between 40 and 60%. It was thus necessary to further purify these β-sitosteryl esters.

### 3.3. Purification of the β-Sitosteryl Esters

The synthesized β-sitosteryl esters were purified using a low-pressure silica column. A glass column (4 cm × 40 cm) packed with 300 mL silica gel (Sigma-Aldrich, Overrijse, Belgium) dispersed in hexane was used to remove β-sitosterol and fatty acids from the β-sitosteryl esters. The first 10 g of reaction product was mixed with eight spoons of silica gel in hexane at 60 °C for 5 min after which the solvent was removed. The silica containing the reaction product was ground to powder and then loaded into the column. Then, 1.8 L of the solvent hexane/ethyl acetate (40:1, *v*:*v*) was allowed to flow through the column and the eluent was collected in tubes of 30 mL. As tested with TLC, the Rf values of β-sitosterol, the fatty acids, and the β-sitosteryl esters in this solvent were 0.00, 0.00 and 0.35, respectively.

Each tube was tested for the presence of β-sitosterol, fatty acids, and β-sitosteryl esters using TLC by applying 10 µL of each test tube to a TLC plate. The plates were developed as described in Section 2.2. and the presence of β-sitosterol, fatty acids, and β-sitosteryl esters on the plate was visualized using a sulfuric acid solution. The tubes contained only β-sitosteryl esters and no β-sitosterol or fatty acids were collected and the solvent was evaporated.

### 3.4. Differential Scanning Calorimetry

The DSC experiments were performed using a DSC Q2000 (TA Instruments, Brussels, Belgium) equipped with an autosampler and nitrogen as a purge gas. Sapphire was used to calibrate the heat capacity and indium was used to calibrate the temperature and enthalpy. An empty pan was used as the reference. About 10–20 mg of melted lipids was sealed into a hermetic aluminum pan (TA Instruments, Brussels, Belgium). The time–temperature program used for the saturated β-sitosteryl esters was as follows: equilibration at 120 °C for 10 min to ensure the complete melting and to erase the crystal memory, followed by cooling at 5 °C/min to 0 °C to obtain the crystallization curve, subsequently holding at 0 °C for 5 min, and finally heating at 5 °C/min to 120 °C to obtain the melting curve. The unsaturated β-sitosteryl esters were subjected to a different time–temperature program. The time–temperature program for P-oleate was equilibration at 60 °C for 10 min, followed by cooling at 5 °C/min to 0 °C to obtain the crystallization curve, and subsequent holding at 0 °C for 5 min, and finally, heating at 5 °C/min to 60 °C to obtain the melting curve. P-linoleate was equilibrated at 40 °C for 10 min, subsequently cooled at 5 °C/min to −60 °C to obtain the crystallization curve, held at −60 °C for 5 min, and finally heated at 5 °C/min to 40 °C to obtain the melting curve. Each measurement was repeated five times.

The parameters derived from the crystallization curve using the Universal Analysis software were T_c_onset_ and T_c_max_. T_c_onset_ was determined by the intersection of the baseline of the crystallization curve with the absolute highest tangent at the high temperature side of the crystallization peak. Concerning the melting curve, four parameters were defined, being T_m_onset_, the peak maximum of the melting peak (T_m_max_) and ΔH_melt_. T_m_onset_ was determined as the intersection of the baseline of the melting curve with the absolute highest tangent at the low temperature side of the melting curve. T_m_max_ was calculated with the Universal Analysis software. In the case of P-laurate, two melting peaks were observed, for which both T_m_max_ were determined. ΔH_melt_, i.e., the amount of heat absorbed during melting, was calculated by the integration of the melting curve using a horizontal baseline at a value equal to the DSC curve at 110 °C for P-stearate and P-palmitate, 90 °C for P-laurate and 38 °C for P-oleate, and −18 °C for P-linoleate. These temperatures mark the high temperature side of the integration range. The intersection of the horizontal baseline with the melting curve at low temperatures was used as the starting point for the integration.

### 3.5. X-ray Diffraction

Time-resolved synchrotron XRD experiments were performed at DUBBLE, the Dutch-Belgian beamline (BM26) at the European Synchrotron Radiation Facility (ESRF; Grenoble, France). SAXS and WAXD patterns were recorded simultaneously using a wavelength, λ, of 1.033 Å. The beamline was equipped with a two-dimensional Pilatus 1 M detector to capture the SAXS signals and a Pilatus 300 K detector to gather the WAXD data. The scattering angles were calibrated with silver behenate and high-density polyethylene standards.

About 10–20 mg of melted lipid was sealed into a hermetic aluminum pan (TA Instruments, Brussels, Belgium) and loaded onto a temperature-controlled sample stage (Linkam HFS 191, Tadworth, UK). The same time–temperature program was used as for the DSC measurements (Section 3.4). During the cooling and heating period, the SAXS and WAXD patterns were collected simultaneously in consecutive 12 s time frames. Each timeframe consisted of 11.985 s measuring time and 0.005 s data saving. The 2D SAXS and WAXD data were azimuthally averaged using the program ConeX [37], normalized to the intensity of the incoming beam measured by a photodiode placed downstream from the sample and corrected for the scattering of an empty setup, including the DSC pan scattering. The WAXD data were further corrected by subtracting a straight line connecting the scattered intensities around 7.7 and 22.0° 2θ. This extra correction approaches the background scattering. In a next step, the WAXD data were normalized to their integrated intensity, which yields scattered intensities due to identical scattering masses. This approach compensates for possible changes in scattering mass by, e.g., melt flow at high temperatures, which might lead to different amounts of material in front of the beam. The WAXD normalization factors were also used to normalize the SAXS patterns for the same effect. For P-stearate, P-palmitate, and P-linoleate, the scattering angle 2θ ranged between 0.48 < 2θ < 7.29° for SAXS and 6.63 < 2θ < 21.9° for WAXD. For P-laurate and P-oleate, the scattering angle 2θ ranged from 0.07 < 2θ < 3.40° for SAXS and 7.72 < 2θ < 28.9° for WAXD. The observed SAXS and WAXD spacings were temperature dependent. The reported spacings of the different crystal structures formed during cooling are those observed at 0 °C. If crystal structures only formed at lower temperatures during cooling or during heating, the spacings were read at the temperature at which they appeared which is reported in Section 2.3.

### 3.6. Polarized Light Microscopy

The PLM experiments were performed on an Olympus BH-2 microscope (Olympus, Berchem, Belgium). A small amount of melted fat was put between two glass slides and submitted to the same time–temperature profile as described in Section 3.4 using a Linkam optical DSC600 hot stage (Linkam Scientific Instruments Ltd., Tadworth, UK), with a liquid nitrogen cooling system. The microscope was equipped with a ProgRes CF color camera (Jenoptik optical systems GmbH, Jena, Germany) to take pictures at regular time intervals in the ProgRes CapturePro 2.10.0.1 software (Jenoptik optical systems GmbH, Jena, Germany). No PLM images could be obtained of the P-linoleate (liquid) crystalline structure due to its low melting temperature. At temperatures below −10 °C, the condensation and crystallization of water in the Linkam optical stage at a temperature below −10 °C blurred the images.

Just as in Kellens et al. (1992), the nucleation rate was determined by counting the number of new nuclei that appeared per time unit in an observation field of 1000 by 1000 µm [20]. As occasionally the nucleation rate increased exponentially during cooling, the average nucleation rate was calculated in a temperature range of 5 °C starting from the formation of the first nucleus.

The crystal growth rate was measured optically by following the increase in spherulite radius as a function of time. Since the crystal growth rate increased slightly with the decreasing temperature the average crystal growth rate during the whole period of crystal growth was calculated for each sample. In the case of P-oleate, many crystals were formed and therefore the crystal growth rate of P-oleate was determined for three spherulites per sample, instead of for each spherulite observed.

## 4. Conclusions

To investigate the influence of the chain length and the degree of unsaturation of the fatty acid in a β-sitosteryl ester on its non-isothermal crystallization and melting behavior, five different β-sitosteryl esters containing different types of fatty acids (stearic (C18:0), palmitic (C16:0), lauric (C12:0), oleic (C18:1), and linoleic acid (C18:2)) were synthesized by enzymatic esterification. The chain length as well as the degree of unsaturation of the fatty acid β-sitosterol ester had an important influence on the crystallization and melting behavior of the β-sitosteryl esters. Table 8 gives a schematic overview of the main conclusions.

The melting temperature and melting enthalpy of the β-sitosteryl esters increased with an increasing fatty acid chain length while they decreased with an increasing degree of unsaturation of the fatty acid chain. The PLM analyses demonstrated that with an increasing degree of unsaturation, the nucleation rate of the β-sitosteryl ester crystals increased, while the crystal growth rate decreased, resulting in a decreasing crystal size. Since the fatty acid chain length did not affect the crystal growth rate of the β-sitosteryl esters, the increasing nucleation efficiency probably resulted in smaller β-sitosteryl ester crystals with an increasing fatty acid chain length. The chain length and the degree of unsaturation of the fatty acid did not influence the morphology of the β-sitosteryl ester crystals, since the saturated β-sitosteryl esters as well as P-oleate formed spherulitic crystals.

As demonstrated by the XRD results, the chain length and especially the degree of unsaturation of the fatty acid also influenced the nanoscale crystal structures formed by the β-sitosteryl esters. It was hypothesized that the saturated β-sitosteryl esters and P-oleate formed a bilayer crystal structure. The SAXS spacings of the β-sitosteryl esters decreased with decreasing fatty acid chain length and with an increasing degree of unsaturation due to a decrease in the lamellar spacings in the bilayer structure. The degree of unsaturation of the fatty acid chain in the β-sitosteryl esters greatly influenced whether or not a metastable or stable mesophase was formed and which type of subcell packing was formed by the fatty acid moieties. A β′ subcell packing was observed in the crystal structure of the saturated β-sitosteryl esters, while the oleic acid moieties formed an α subcell packing in the P-oleate crystal structure.

## Figures and Tables

**Table 1:** Onset temperature (T_c_onset_) and peak maximum of the crystallization curve (T_c_max_), onset temperature (T_m_onset_) and peak maximum (T_m_max_) of the melting curve, melting enthalpy (ΔH_melt_) and difference between the T_c_onset_ and T_m_onset_ (ΔT) of the different β-sitosteryl esters. Values show the mean ± standard deviation of 5 repetitions.

β-Sitosterol Ester	T_c_onset_ (°C)			T_c_max_ (°C)			T_m_onset_ (°C)			T_m_max_ (°C)			ΔH_melt_ (J/g)			ΔT (°C)		
P-stearate	73.4	±	0.4	73.6	±	0.6	89.5	±	0.1	92.1	±	0.1	88.8	±	7.2	16.1	±	0.4
P-palmitate	65.8	±	1.0	65.6	±	0.9	86.0	±	0.3	89.1	±	0.1	87.6	±	1.1	20.2	±	1.2
P-laurate	48.1	±	2.8	47.3	±	1.9	61.9	±	3.6	69.9	±	0.0	58.9	±	3.9	10.5	±	1.2
										78.2	±	0.1						
P-oleate	18.8	±	0.2	15.2	±	0.4	18.5	±	2.2	27.3	±	0.2	45.1	±	5.5	−0.9	±	2.2
P-linoleate	43.4	±	1.4	−45.1	±	1.8		-		−41.9	±	1.8	2.3	±	0.5		-	

**Table 2:** Small angle X-ray scattering (SAXS) and wide angle X-ray diffraction (WAXD) spacings of the new peaks appearing during the cooling and heating of P-stearate with an indication of whether their intensity was relatively weak (w) or strong (s) and with their identification (ID) as an order reflection of bilayer 1 or bilayer 2 (with an apostrophe) or as related to the packing of β-sitosterol moieties (SI) or the β′ subcell packing of the stearic acid moieties (β′) or unidentified (UI). The intensity of the spacings that emerged during the heating was weak. λ = 1.033 Å. The error on the scattering angle is 0.01°2θ.

P-Stearate	Cooling: From 71.8 °C				Heating: 86.2–93.2 °C			Heating: 93.2–100.6 °C		
	2θ (°)	d (Å)	w/s	ID	2θ (°)	d (Å)	ID	2θ (°)	d (Å)	ID
SAXS	1.00	59.2	s	1				1.04	56.9	1′
	1.99	29.7	s	2				2.07	28.6	2′
	2.98	19.9	s	3				3.11	19.0	3′
	3.97	14.9	w	4						
	4.97	11.9	w	5						
	5.95	9.95	w	6						
	6.95	8.52	w	7						
	7.21	8.21	w	UI						
WAXD	7.86	7.53	s	8	6.05	9.78	UI	9.92	5.97	10′
	8.10	7.31	w	SI	11.9	4.97	UI	10.1	5.86	SI
	8.75	6.76	w	9	15.2	3.89	16	11.0	5.38	UI
	9.10	6.50	w	UI	15.5	3.82	β′	11.7	5.06	UI
	9.80	6.04	s	10				12.4	4.77	DI
	10.5	5.64	w	SI				13.9	4.26	UI
	10.7	5.53	w	11				15.9	3.72	16′
	11.4	5.19	w	UI						
	12.3	4.81	w	SI						
	12.7	4.66	w	13						
	13.1	4.52	w	UI						
	13.6	4.35	w	14						
	14.1	4.20	w	β′						
	14.4	4.11	w	15						
	15.4	3.84	s	16						
	16.6	3.57	w	UI						

**Table 3:** Small angle X-ray scattering (SAXS) and wide angle X-ray diffraction (WAXD) spacings of the new peaks appearing during the cooling and heating of P-palmitate with an indication of whether their intensity was relatively weak (w) or strong (s) and with their identification (ID) as an order reflection of bilayer 1 or bilayer 2 (with an apostrophe) or as related to the packing of β-sitosterol moieties (SI) or the β′ subcell packing of the palmitic acid moieties (β′) or unidentified (UI). The intensity of the spacings that emerged during the heating was weak. λ = 1.033 Å. The error on the scattering angle is 0.01 °2θ.

P-Palmitate	Cooling: from 64.7 °C				Heating: 70.3–91.3 °C			Heating: 91.3–95.6 °C		
	2θ (°)	d (Å)	w/s	ID	2θ (°)	d (Å)	ID	2θ (°)	d (Å)	ID
SAXS	1.04	56.9	s	1	4.12	14.4	4	1.1	53.8	1′
	2.08	28.5	s	2	5.15	11.5	5	2.18	27.2	2′
	3.12	19.0	s	3				3.27	18.1	3′
	6.12	9.67	s	6						
	6.38	9.28	w	UI						
	6.81	8.69	w	UI						
	7.33	8.07	w	7						
WAXD	7.75	7.64	w	UI	11.8	5.02	UI	7.92	7.47	UI
	7.92	7.47	w	8	14.5	4.08	UI	9.34	6.34	9′
	8.63	6.86	w	SI				9.63	6.15	UI
	9.28	6.38	w	UI				10.1	5.86	SI
	9.75	6.07	s	10				11.6	5.10	11′
	10.5	5.64	w	SI				12.3	4.81	SI
	12	4.93	s	12				13.7	4.32	13′
	12.3	4.81	w	SI						
	12.6	4.70	w	UI						
	12.9	4.59	w	13						
	14.2	4.17	s	14						
	15.3	3.87	w	SI						
	15.7	3.77	w	β′						
	16.4	3.61	w	UI						
	17.4	3.40	w	UI						
	17.9	3.31	w	18						

**Table 4:** Small angle X-ray scattering spacings of the new peaks appearing during the cooling and heating of P-laurate with an indication of whether their intensity was relatively weak (w) or strong (s) and with their identification (ID) as an order reflection of bilayer 1, bilayer 2 (with an apostrophe) or bilayer 3 (with double apostrophe), or as related to the packing of β-sitosterol moieties (SI) or the β′ subcell packing of the lauric acid moieties (β′) or unidentified (UI). λ = 1.033 Å. The error on the scattering angle is 0.01 °2θ.

P-Laurate	Cooling: From 47.4 °C				Heating: 44.8–69.1 °C				Heating: 69.1–81.0 °C			
	2θ (°)	d (Å)	w/s	ID	2θ (°)	d (Å)	w/s	ID	2θ (°)	d (Å)	w/s	ID
SAXS	1.14	51.9	s	1	1.22	48.5	s	1′	1.1	53.8	s	1″
	2.28	26.0	s	2	2.44	24.3	s	2′	2.18	27.2	s	2″
WAXD	7.85	7.54	w	7	8.11	7.30	w	UI	8.06	7.34	w	UI
	8.49	6.97	w	SI	9.74	6.08	w	8′	9.13	6.48	w	UI
	9.21	6.43	w	8	9.89	5.98	s	UI	9.80	6.04	s	9″
	9.94	5.95	s	9	10.9	5.43	w	9′	10.0	5.92	s	SI
	10.2	5.80	w	SI	13.8	4.29	s	UI	10.8	5.47	w	10″
	11.7	5.06	w	UI	15.6	3.79	w	β′	11.5	5.16	w	UI
	11.9	4.97	w	UI					11.7	5.07	s	UI
	12.1	4.89	s	11					12.0	4.94	w	11″
	12.3	4.81	w	SI					12.5	4.75	s	SI
	12.6	4.70	w	UI					13.0	4.55	w	12″
	13.5	4.38	w	UI					13.6	4.37	w	UI
	13.6	4.35	w	UI					13.7	4.31	s	UI
	13.9	4.26	s	12					14.0	4.23	s	UI
	14.1	4.20	s	β′					14.3	4.14	w	13″
	14.5	4.08	w	UI					14.8	4.01	w	UI
	14.7	4.03	w	13					15.1	3.92	w	UI
	15.4	3.86	w	UI					15.3	3.88	w	14″
	15.8	3.75	w	14					15.8	3.75	w	UI
	16.2	3.66	w	UI								

**Table 5:** Small angle X-ray scattering spacings of the new peaks appearing during the cooling of P-oleate and P-linoleate with an indication of whether their intensity was relatively weak (w) or strong (s) and with their identification (ID) as an order reflection of the bilayer structure or as related to the packing of the β-sitosterol moieties (SI), the α subcell packing of the oleic acid moieties (α) or the liquid crystals (LC) or unidentified (UI). λ = 1.033 Å. The error on the scattering angle is 0.01 °2θ.

P-Oleate					P-Linoleate (From −20.7 °C, in SAXS)			
**From 14.7 °C**	**2θ (°)**	**d (Å)**	**w/s**	**ID**	**2θ (°)**	**d (Å)**	**w/s**	**ID**
SAXS	1.62	36.6	s	LC	1.60	37.1	s	LC
**From 11.8 °C**	**2θ (°)**	**d (Å)**	**w/s**	**ID**				
SAXS	1.03	57.5	w	1				
	2.14	27.7	s	2				
	3.25	18.2	w	3				
WAXD	7.89	7.50	w	8				
	8.06	7.34	w	SI				
	9.89	5.98	s	10				
	11.1	5.33	w	UI				
	11.6	5.10	m	12				
	12	4.93	w	SI				
	13.7	4.32	s	14				
	14.3	4.14	w	α				
	15.3	3.87	w	SI				

**Table 6:** Sterol composition (%) of β-sitosterol (average of two measurements, the error is 0.2%).

Sterol	
β-sitosterol	80.9
β-sitostanol	12.9
campesterol	6.2

**Table 7:** Fatty acid composition (%) of oleic acid (average of three measurements, the error is 0.2%).

Fatty Acid	
C16:0	3.0
C18:0	4.0
C18:1	91.2
C24:1	1.7

**Table 8:** Schematic overview of the main conclusions.

Effect of Fatty Acid Chain in PEs	Increasing Chain Length	Increasing Degree of Unsaturation
**T_m_onset_**	↗	↘
**∆H_m_**	↗	↘
**Nucleation rate**	↗	↗
**Crystal growth rate**	No effect	↘
**Spherulite size**	↘	↘
**Lamellar spacing**	↗	↘
**Type of subcell packing**	No effect, always β′	P-oleate: α
**Formation of liquid crystals (LC)q**	No	P-oleate: metastable LC P-linoleate: stable LC
